# A rollercoaster of emotions: An integrative review of emotions and its impact on health professional students' learning in simulation‐based education

**DOI:** 10.1002/nop2.1100

**Published:** 2021-10-21

**Authors:** Anine Madsgaard, Hilde Smith‐Strøm, Irene Hunskår, Kari Røykenes

**Affiliations:** ^1^ VID Specialized University Fyllingsdalen Norway

**Keywords:** emotions, learning, simulation

## Abstract

**Aim:**

Simulation‐based education establishes a specific learning environment capable of activating emotions before, during and after the task. Research has identified stress and anxiety related to simulation. However, the role of various emotional experiences in a simulation that favour learning is still unclear. This review describes, interprets and synthesizes the current research findings on health professional students' experience of emotions and the effects on student learning in simulations.

**Design:**

This study design was guided by integrative review method.

**Methods:**

Databases were systematically searched for articles. 9,323 records were screened and 16 studies met the inclusion criteria. The study protocol was reported in Prospero.

**Results:**

Three themes emerged from the analysis: (a) simulation as a fearful and stressful situation, (b) variability in emotions experienced during simulation as a rollercoaster of emotions and (c) emotions wide‐ranging effects on students' learning in the simulation.

## 1 INTRODUCTION

Emotions can influence memory, attention, cognitive and metacognitive thinking strategies, judgement, decision‐making, cognitive resources and both intrinsic and extrinsic motivation (Artino et al., 2012; Gluck et al., 2016). Research into how emotions affect learning has increased in the past decade (Pekrun, 2019; Pekrun & Linnenbrink‐Garcia, 2014). However, the ability of the context and learning activities to sway students' emotions have not been investigated thoroughly, especially for simulated learning. Simulation‐based education (SBE) is a learning activity in which the clinical work environment is simulated in the laboratory. Simulation has attracted attention in health‐care education and is an important method for teaching clinical skills in a safe learning environment (Campbell & Daley, 2018; Levine et al., 2013; Motola et al., 2013). Education in nursing, medicine, pharmacology, physiotherapy, dentistry and occupational therapy are some of the health areas currently using SBE (Bethea et al., 2014; Blackstock & Jull, 2007; Harder, 2010; Hattingh et al., 2018; Perry et al., 2015).

By working in teams during SBE, students learn to manage patient situations in a realistic environment but away from real patients (Campbell & Daley, 2018; Jeffries, 2020). The benefits for health professional students are the opportunity to practise in a safe environment and make mistakes without harming patients. Teachers can adapt the situations to achieve specific learning outcomes. In an academic situation, emotions are often provoked by the learning environment itself, and research has shown that emotions can impact learning (Pekrun & Linnenbrink‐Garcia, 2014). Because students are highly activated both mentally and physically, SBE is a specific learning environment that can lead to the activation of emotions before, during and after the task.

Historically, research on cognition and emotions has been conducted separately, although there has been a shift towards a more integrative approach to provide a holistic understanding of students' learning processes. Studies have focused on student learning and reflection during simulation (Cant & Cooper, 2010, 2017; Hegland et al., 2017; Husebo et al., 2013). Student learning outcomes include acquisition of patient care skills and clinical competency in addition to improved knowledge, consider alternative solutions to a clinical problem and critical thinking abilities. SBE has been found to boost student self‐confidence and satisfaction (Cant & Cooper, 2010; Issenberg et al., 2005; Olaussen et al., 2020). However, despite these learning outcomes and student satisfaction, students participating in SBE also report anxiety and stress (Al‐Ghareeb et al., 2017; Cantrell et al., 2017).

There is a need to study the role of emotions in different contexts to understand how emotions can affect student learning (Artino et al., 2012; Pekrun, 2019). Health education has ignored the important role emotional experiences have for learning (LeBlanc et al., 2015). Given the current importance of SBE for health professional students, a greater understanding of the importance of emotions to learning in this context is needed.

This review describes, interprets and synthesizes the current research findings on health professional students' experience of emotions and the effects on student learning in simulations.

### 1.1 Background

#### 1.1.1 Simulation‐based education

Simulation‐based education usually follows a template (Jeffries, 2020) starting with a briefing session which students are informed about the patient's situation and learning goals and are allowed to become comfortable with the equipment. In second session, the scenario unfolds, and students become engaged and active in solving the patient's problems. Finally, a debriefing session provides the opportunity for students to reflect on and discuss their actions. Debrief has been identified as the aspect in which most learning occurs (Dufrene & Young, 2014; Levett‐Jones & Lapkin, 2014). Debriefing provides the opportunity for complex learning by identifying problems that occurred during the simulation and how these were solved, making comparisons and explaining their actions as a way to demonstrate their learning.

#### 1.1.2 Learning in SBE

Students add new information to their already existing knowledge while learning. This reconstruction of knowledge takes place through the interplay between cognition, emotions and social dimensions (Biggs & Collis, 2014; Illeris, 2015). This process is also relevant to SBE. During simulation, students are asked to apply their existing knowledge and skills in a new setting. To do so students create new connections with varied solutions and dilemmas, and this process challenge them to learn on a deeper level. The intention of SBE is to facilitate the extension of the current understanding into new contexts, in this case, health professional practice. Through this process, learning is extended to more abstract and complex levels (Biggs & Collis, 2014).

#### 1.1.3 Academic emotions in SBE

**FIGURE 1:**
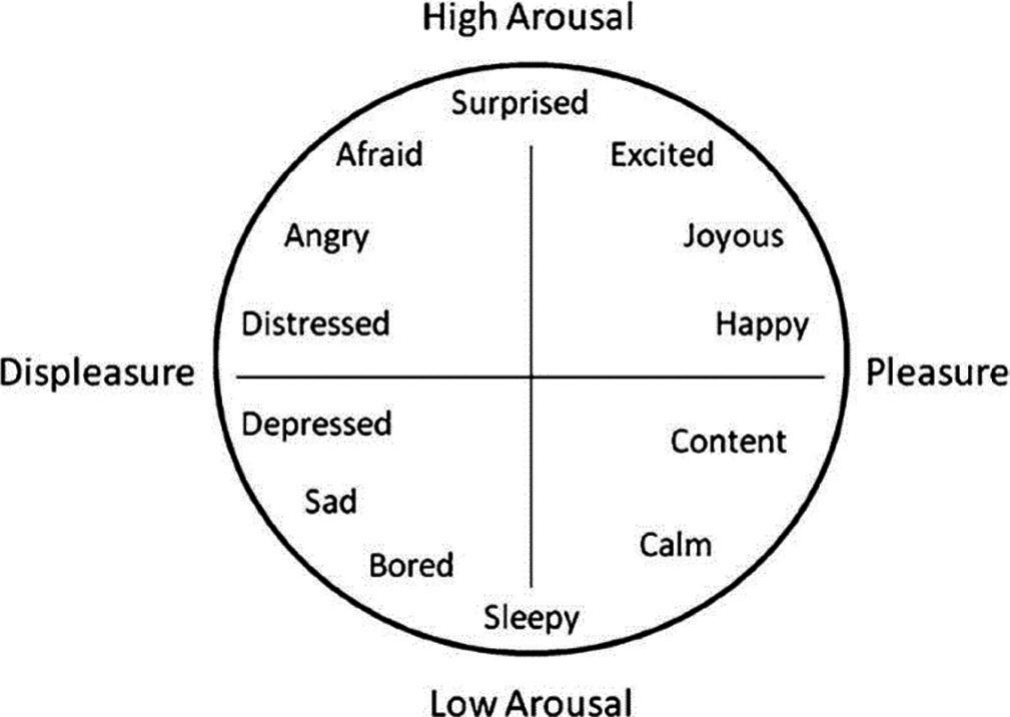
A representation of the circumplex model of affect. The horizontal axis representing the valence dimension and the vertical axis representing arousal (Barrett & Russell, 2014)

Participating in SBE can activate students both mentally and physically. This activation can trigger academic emotions before, during and after SBE. Academic emotions are those that can arise in different academic settings, and emotions reflect a heterogeneous experience that encompasses a wide variety of psychological phenomena (Barrett, 2004). Emotions are explored and expressed widely, and can be categorized as positive emotions, such as happiness, hope, joy and satisfaction, or negative emotions such as fear, anxiety, sadness, shame and disgust (Pekrun & Linnenbrink‐Garcia, 2014). The circumplex model (CM) of affect classifies emotions into two dimensions: valance and arousal (Posner et al., 2005) (Figure 1). Valence is the experience of a situation as either positive and pleasant or negative and unpleasant. Arousal is the human experience of being either physiologically activated or deactivated in a situation (Barrett & Russell, 2014).

In any given learning situation, valence and arousal dimensions occur in combinations (Roth & Walshaw, 2019). High arousal activates the brain and this improves attention and memory. Valence has a complex impact on learning; that is, positive emotions can broaden attention and cognition, and negative emotions can promote deeper analytic processes and sharpen and detailed memory (LeBlanc et al., 2015).

A range of emotions is essential for engaging and completing an academic task. Emotions can influence the learning process in many ways by impacting cognitive resources, long‐term memory, cognitive and metacognitive thinking strategies, and motivational processes (Artino et al., 2012). Emotions are related to the perceptions associated with academic activity (e.g. anxiety, joy or boredom) and to the feeling of success or failure after completion of an academic activity (e.g. pride or shame).

Emotions can be activated when students experience cognitive incongruity, for example, when students discover they are unable to solve a learning task problem and thereby experience surprise, frustration or confusion. Such emotions can have a positive effect on knowledge acquisition (Vogl et al., 2019). Emotions can also be elicited by the content within learning material, such as being sad if the manikin in the simulation dies. Because learning activities take place in a social setting, students can experience social achievement emotions such as shame, for example, if they demonstrate their lack of knowledge in front of their peers and teachers (Pekrun & Linnenbrink‐Garcia, 2014).

Negative emotions such as stress and anxiety occur frequently in simulation settings and are thought to interfere with learning (Al‐Ghareeb et al., 2017; LeBlanc et al., 2015). Limited research has identified positive emotions in simulation settings. One study explored students' emotional experiences during a simulation in continuing education and found that the students experienced mainly positive emotions such as interest, enjoyment of learning and cheerfulness both before and after the simulation (Keskitalo & Ruokamo, 2017).

Because emotions can be triggered by activity and social interactions, it is assumed that students can experience varied and multiple emotions during SBE. It has been found that emotional experiences can both inhibit and promote learning (Pekrun & Linnenbrink‐Garcia, 2014). Previous research in SBE has focused mainly on the presence of student's anxiety (Al‐Ghareeb et al., 2017). For educators to develop and deliver comprehensive SBE, further understanding of students' emotions and their effect on learning is needed. This integrative review was performed to summarize the current state of knowledge of emotions and emotions' effects on health professional students' learning SBE.

## 2 METHODS

Briefly, this review followed the integrative review methodology described by Whittemore and Knafl (2005). A multidimensional approach for searching the research literature by synthesizing quantitative and qualitative findings (Noyes et al., 2019). The approach included four phases to provide a full and comprehensive understanding of current research on students' emotions and learning in SBE.

The study protocol was reported in Prospero (CRD 42018107758).

### 2.1 Study design

The review was performed following the four stages of Whittemore and Knafl (2005): problem identification, systematic search of the literature, evaluation and analysis of data.

### 2.2 Search

The main electronic search was conducted in June 2018, using databases included in the Cumulative Index to Nursing and Allied Health Literature, Education Source, MEDLINE, SveMed+, PsycInfo, Science Direct and Educational Resources Information Center. The search terms included words related to emotions found in the CM combined with words related to different health students' professions. The main search terms used were Simulation, Scenario‐based simulation, Simulation‐based education, Emotions, Academic emotions, Occupational therapy, Dental, Medical, Nursing, Pharmacy and Public health students. The last updated search was conducted in April 2021. The researchers (AM, HSS, IH and KR) discussed these terms to ensure the correct terms were used and combined. Because of the integrative review, no limitations regarding study design or methods were imposed in the searches (Whittemore & Knafl, 2005). The searches also had no limitations regarding languages.

The first author (AM) performed hand searches of articles published in relevant journals and the references included in full‐text articles. To ensure as extensive a search as possible, integrative reviews often perform searches of multiple sources rather than relying only on electronic databases.

The inclusion and exclusion criteria (Table 1) were compiled after first considering the aim of this review; these criteria guided the final decision about inclusion in the review.

**TABLE 1:** Inclusion and exclusion criteria

Inclusion	Exclusion
Health profession students, all professions	Young adults under age 18 years Professional health workers
Simulation‐based learning Scenario‐based learning Simulation‐based education	Virtual and gaming simulation
Emotions, learning and simulation	Self‐efficacy Anxiety and stress measuring Simulation as stress a reducer in practice
Adult education	
Published after 1999	
English and Scandinavian languages	
Peer‐ reviewed articles Dissertations	Books and book chapters Conference proceedings Short papers Grey literature Reports Editorials Reviews

### 2.3 Data evaluation and results of the search

The search results were imported from EndNote to the screening tool Rayyan. Four researchers were engaged in the screening process. We used the following process for quality assurance of the screening process. The research group had a common understanding of the study aim and the inclusion and exclusion criteria. All four researchers screened 10 untargeted abstracts for calibration. AM, KR, HSS and IH independently screened 200 abstracts each. Rayyan was opened for double blinding of the 800 abstracts. The research team discussed disagreements and discrepancies in meetings. Inclusion and exclusion criteria were compiled by all researchers. First author screened the remaining 8,523 abstracts and titles alone. There was no disagreement about full‐text inclusion. Ninety‐three full‐text articles and dissertations were assessed for eligibility. All four researchers read the full‐text material and independently included or excluded articles. After discussion, 16 studies were included in the analysis.

**FIGURE 2:**
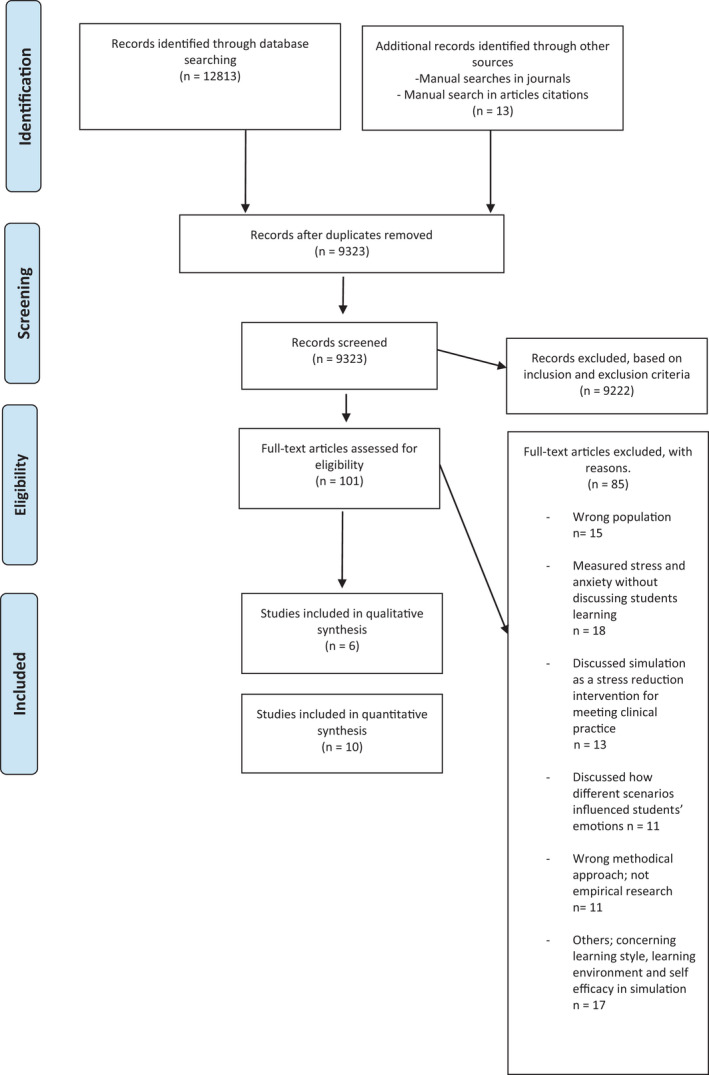
Flowchart

Figure 2 describes the screening process using a PRISMA flow chart.

### 2.4 Data analysis

Three authors (AM, KR and HSS) conducted the analyses individually. All authors met to discuss the data to ensure their credibility and to obtain consensus about the interpretation of the individual themes that emerged during analysis. The analytic process focused on identifying common and unusual patterns, variations, comparisons and relationships. This process allowed us to conduct the analysis to meet the aim of this review regardless of the research methodology or study design (Whittemore & Knafl, 2005).

We obtained comprehensive insights into the data using the following phases. First, reading the studies and especially the results provided us with an overall impression of the data and a sense of the whole. Second, the data were open coded and clustered thematically. We next compared the themes within studies to contrast patterns within each study. Finally, the themes were compared and contrasted between studies in an integrative matrix synthesis (Noyes et al., 2019; Whittemore & Knafl, 2005).

### 2.5 Critical Appraisal Skills Programme

The Critical Appraisal Skills Programme (CASP, 2013) was used to appraise the methodological quality of the included articles systematically. To minimize bias, three researchers were involved (AM, KR and HSS). To perform an in‐depth appraisal and to adhere to the methodological appraisals, two summary matrices were utilized: CASP for quantitative studies and CASP for qualitative studies (Tables 2 and 3 respectively). The studies were evaluated according to their presentation of the design, methods, ethical quality, analyses and discussion. The quality of the studies was evaluated to identify the invalid and highly valid studies.

**TABLE 2:** CASP Quantitaive

	1	2	3	4	5	6	7	8	9	10
Al‐Ghareeb et al. (2019)	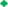	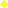	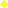	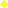	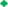	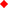	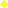	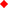	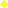	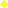
Beischel (2013)	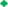	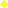	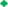	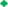	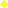	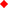	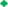	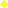	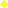	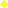
Cato (2013)	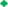	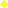	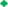	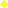	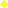	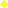	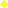	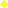	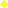	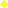
Fraser et al. (2012)	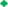	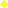	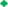	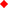	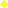	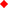	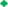	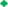	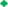	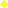
Fraser et al. (2014)	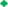	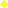	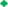	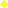	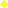	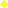	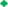	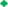	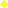	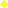
DeMaria et al. (2010)	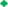	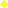	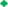	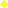	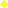	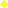	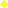	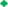	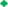	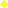
DeMaria et al. (2016)	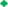	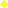	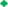	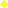	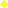	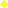	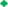	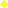	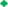	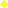
Fraser and McLaughlin (2018)	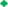	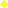	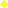	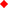	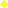	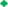	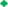	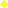	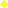	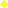
Schlairet et al. (2015)	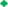	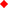	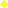	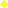	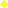	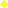	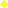	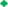	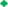	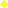
Tremblay et al. (2017)	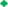	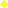	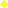	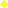	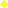	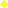	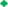	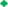	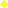	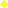

Note

(1) Design, purpose/aim and background.

(2) Methods, sufficiently described, location and dates of data collection.

(3) Data analysis, description of methods used for data analysis, verifying data, calculating the response rate.

(4) Sample selection, sample size calculation, representativeness.

(5) Research tool development, description of the population.

(6) Administration of tool, who approached potential participants, number of contacts provided.

(7) Ethical quality, approval, consent.

(8) Results, response rate reported, respondents accounted for, results clearly presented.

(9) Discussion, results summarized, strengths and limitations, the generalizability of results discussed.

(10) Overall rating: high quality, medium quality, low quality, exclusion.

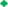
 = High, all/almost all criteria met.

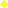
 = Medium, some of the criteria are not met or not satisfactorily describe.

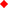
 = Low, few or no criteria are met or satisfactorily described.

**TABLE 3:** CASP qualitative

	1	2	3	4	5	6	7	8	9	10	11	12
Behrens et al. (2019)	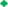	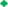	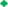	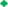	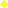	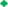	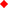	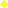	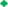	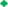	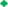	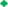
Holt (2017)	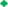	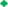	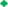	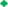	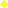	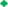	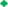	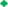	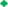	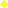	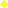	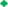
Groot et al. (2020)	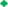	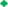	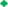	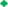	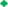	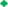	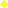	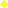	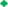	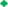	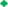	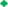
Ko and Choi (2020)	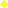	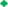	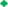	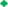	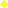	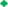	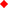	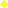	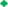	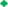	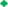	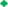
Najjar et al. (2015)	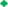	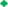	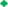	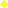	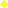	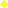	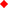	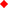	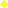	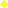	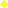	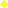
Walton et al. (2011)	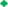	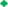	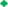	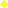	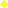	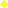	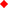	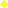	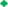	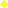	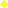	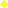

Note

(1) A clear statement of the aim.

(2) Was a qualitative methodology appropriate?

(3) Is it worth continuing?

(4) Was the design appropriate to address the aims of the research?

(5) Was the recruitment strategy appropriate?

(6) Were the data collected in a way that addressed the research issue?

(7) Has the relationship between the researcher and participants been addressed?

(8) Have ethical issues been taken into consideration?

(9) Was the analysis sufficient?

(10) Is there a clear statement of findings?

(11) How valuable is the research?

(12) Overall rating: high quality, medium quality, low quality, exclusion.

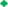
 = High, all/almost all criteria met.

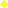
 = Medium, some of the criteria are not met or not satisfactorily describe.

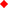
 = Low, few or no criteria are met or satisfactorily described.

## 3 RESULTS

Table 4 describes the purpose, sample, methods and design used to report emotions and learning in addition to the study findings from the included studies.

**TABLE 4:** Summary of included studies

Authors, year, location	Purpose	Participants, sample	Study design, method and measurement tools	Study findings	
				Emotions	The learning process and learning outcome
1. Al‐Ghareeb et al. (2019) Australia	Investigated anxiety during the simulation and the effect of anxiety on clinical performance.	*n* = 33 Nursing students	Mixed method: Questionnaire Heart rate measuring Performance rating	Anxiety Psychological anxiety was higher at the end of the simulation. Physiological anxiety and heart rate were higher at the start than at the end.	Relationship between physiological anxiety and clinical performance was not significant. The result indicated that low‐level anxiety led to optimal performance.
2. Beischel (2013) USA	Explored students' perceptions on how simulation affects anxiety and learning.	*n* = 124 Nursing students	Mixed method: Survey questionnaire Self‐reported questionnaire Semi‐structured group discussion	Anxiety	Dissonance found between quantitative and qualitative data. Students reported increased levels of anxiety which negatively affected their learning. Quantitative data indicated anxiety did not mediate cognitive learning outcomes.
3. Behrens et al. (2019) Chile	Exploring undergraduate student's achievement emotions during ward round simulation: a mixed‐method study	*n* = 53 Medical students	Mixed method: Achievement emotions questionnaire (AEQ) Postgraduate Ward round simulation assessment tool (PgWRS) Focus group	Positive emotions enjoyment, pride and hope obtained the highest scores. Anxiety was identified. Feeling of enjoyment, and pride during the simulation. Pride and happiness, shame and frustration after ended experience.	The positive emotions of pride and enjoyment were experienced as a positive drive for learning. The same holds for a moderate level of anxiety. Emotions did not correlate significantly with performance.
4. Cato (2013) USA	Explored students' experience in a simulation. Identified causes for anxiety, and how anxiety affected learning.	Quantitative *n* = 178 Qualitative *n* = 9 Nursing students	Mixed method: Self‐composed survey Focus group	Anxiety Physiological reaction such as sweating and shaking.	Memory loss and decreased ability to focus.
5. DeMaria et al. (2010) USA	Determined if a simulation with added emotional stress could provoke anxiety and, if so, whether participants' learning was influenced by emotional stress.	*n* = 25 Medical students	Quantitative Intervention study with a control group: Survey Observation ranking Heart rate monitor Knowledge measured by written test score and final mega code	Stress The intervention group experienced greater anxiety than the control group on both anxiety score and heart rate.	The intervention group achieved higher practical competency examination than the control group.
6. DeMaria et al. (2016) USA	Described physiological and biochemical stress response induced by simulated patients' death and impact on long‐term retention of advanced cardiovascular life support knowledge and skills.	*n* = 26 Medical students	Quantitative study: During simulation: Heart rate monitor Biomarker response After six months: Written exam and rating observing	Stress Increased heart rate, saliva cortisol and DHEA during the simulation compared with baseline for all participants. Increased heart rate response with participants in the death group.	No difference on long‐term knowledge or skills.
7. Fraser et al. (2012) Canada	Explored relationships between emotion, cognitive load and diagnostic performance.	*n* = 84 Medical students	Quantitative Survey study: Survey questionnaire Performance observation	Identified two emotional components, invigoration and tranquillity, both associated with cognitive load.	A significant negative association between cognitive load and the odds of subsequently identifying trained murmur.
8. Fraser et al. (2014) Canada	Stimulating emotions during simulation and impact on cognitive load and learning.	*n* = 116 Medical students	Quantitative, Prospective Randomized trial: Survey questionnaire Performance in OSCE exam	More negative emotions in groups where the simulated patient died.	Students reporting negative emotions reported a higher cognitive load and were less likely to be rated as competent to diagnose and manage a patient 3 months later.
9. Fraser and McLaughlin (2018) Canada	Understand learners' emotional experience during each phase of the simulation session.	*n* = 174 Medical students	Quantitative: Survey	Students' experience of tranquillity dropped from pre‐scenario to post‐scenario and returned to baseline level after debriefing. Post‐scenario cognitive load was rated to be moderately high, and scores increased after debriefing.	Cognitive load was associated with simultaneous measures of emotions.
10. Groot et al. (2020) Netherland	To understand the psychosocial and educational impact of simulation	*n* = 11 Medical students	Qualitative Phenomenological interviews	Stress Uncertainty Disappointment	Stress turned simulation into a motivating educational experience.
11. Holt (2017) USA	Explored affective learning from the perspective of nursing students participating in a simulation.	*n* = 25 Baccalaureate nursing students	Qualitative Phenomenological inquiry, Individual interviews	Anxious about not knowing. Excited by growing and developing. Enjoyed learning. Confused. Pressured by being observed.	Learned to communicate and work in teams, life‐long learning, confronting ethical issues, empathy for patients, families and peers and increased self‐confidence.
12. Ko and Choi (2020) South Korea	Debriefing Model for Psychological Safety in Nursing Simulations: A Qualitative Study	*n* = 23 Undergraduate nursing students	Qualitative, in depth interviews	Stress, fear, embarrassed, nervous, disappointed.	In attempt to reduce stress and nervousness students prepared for simulation beforehand which were helpful for learning Experienced positive effects of stress on learning
13. Najjar et al. (2015) USA	Described nursing students' experience in high fidelity simulation and developed a model that explicates the experience.	*n* = 26 Nursing students	Qualitative, Grounded Theory, Focus group interviews Semi‐structured interviews	Sigh of relief, high anxiety, nerve wracking, overwhelmed, uncomfortable with manikins, frustrated	Learning from others, reflecting, gaining confidence, gaining experience.
14. Schlairet et al. (2015) USA	Explored the impact of simulation on emotion and cognitive load.	*n* = 40 Nursing students	Quasi‐experimental pilot study: Survey Observation	Two emotional states identified: pleasant activation and pleasant deactivation components. Cognitive load following simulation was high	Identified a negative but statistically non‐significant effect of cognitive load on assessment performance.
15. Tremblay et al. (2017) Canada	Determined whether Simulated Clinical Immersion imposes greater extraneous cognitive load and stress than simulated patients without environment.	*n* = 143 Undergraduate pharmacy students	Mixed Method: Survey Focus groups interview	Stress Performance anxiety	The physical environment of SCI was stressful and probably hindered learning.
16. Walton et al. (2011) USA	Understand how students learn with simulation and identify basic social processes.	*n* = 26 Baccalaureate nursing students	Qualitative, Grounded theory: In‐depth interviews Focus group	Feeling disorganized, feeling uncomfortable and anxious, joking, struggling, unsure, disappointment, devastation, lack of confidence and fear.	Learning process: Difficulties thinking and doing. Learning outcomes: Priorities, administering medication, identified areas for improvement, analyse patients' situations and understand the role of nurses.

The academic emotions described in the results were evoked by the simulation learning task itself. Emotions vary during the learning activity and were found to unfold from negatively to positively loaded. Three themes emerged from the analysis: (a) simulation as a fearful and stressful situation, (b) variability in emotions experienced during simulation as a rollercoaster of emotions and (c) emotions wide‐ranging effects on students' learning in the simulation.

The research findings are presented in detail in the following sections.

### 3.1 Theme 1: Simulation as a fearful and stressful situation

Our analysis showed that negatively loaded emotions were mentioned frequently in the literature. Nine studies investigated anxiety (Al‐Ghareeb et al., 2019; Behrens et al., 2019; Beischel, 2013; Cato, 2013; Fraser et al., 2012; Fraser & McLaughlin, 2018; Holt, 2017; Najjar et al., 2015; Walton et al., 2011). Four studies investigated stress (Demaria et al., 2010, 2016; Groot et al., 2020; Tremblay et al., 2017). Students have reported feeling anxious, disappointed, uncomfortable, confused, nervous, fearful, devastated and angry during the simulation (Holt, 2017; Ko & Choi, 2020; Najjar et al., 2015; Walton et al., 2011).

Several studies highlighted the reasons for anxiety and stress associated with simulations (Beischel, 2013; Cato, 2013; Groot et al., 2020; Holt, 2017; Ko & Choi, 2020; Najjar et al., 2015; Tremblay et al., 2017; Walton et al., 2011). Fear of the unknown and being evaluated, uncertainty about treatment options and feeling unprepared for the simulation setting are important explanations. Inexperience, self‐doubt, fear of performing in front of peers and teachers, fear of being on camera and anxiety about potential for making mistakes are triggers. Several studies also examined anxiety about the high‐fidelity unfamiliar environment, stress related to the equipment not working as intended and the manikin's death. (Demaria et al., 2010, 2016; Fraser et al., 2014; Tremblay et al., 2017; Walton et al., 2011).

The students' different roles in a simulation setting trigger negatively loaded emotions. The active leading student role or being a team leader generates more stress, anxiety and cognitive overload than having an observer role (Beischel, 2013; Cato, 2013; Fraser et al., 2012; Schlairet et al., 2015; Tremblay et al., 2017; Walton et al., 2011) because students are put on the spot, must perform in front of teachers and peers, and are anxious about receiving feedback in the debriefing.

### 3.2 Theme 2: Variability in emotions experienced during simulation as a rollercoaster of emotions

Emotions vary before, during and after the simulation (Tremblay et al., 2017; Walton et al., 2011). Anxiety, being alert, feeling insecure or disorganized and joking to mask fear are predominant at the beginning of a simulation. Stress, embarrassment, confusion, enjoyment and pride are evident during the unfolding of the scenario. The various reported emotional outcomes after the end of the simulation range from fear, anxiety, shame, frustration, confidence, calm, nervousness, disappointment, surprise and worry, to enjoyment, excitement, happiness, pride and feeling challenged (Behrens et al., 2019; Fraser et al., 2012; Fraser & McLaughlin, 2018; Groot et al., 2020; Holt, 2017; Ko & Choi, 2020; Schlairet et al., 2015; Walton et al., 2011). Students also report feeling a “sigh of relief” after the scenario has ended (Holt, 2017; Walton et al., 2011).

The research also described tranquillity and invigoration (Fraser et al., 2012; Fraser & McLaughlin, 2018; Schlairet et al., 2015). Invigoration was predominant and suggests that students can experience simulation as a pleasant and activating learning activity. Fraser and McLaughlin (2018) found that tranquillity seems to increase during the debriefing, which suggest that students feel comfortable and relaxed during the debriefing.

### 3.3 Theme 3: Emotions wide‐ranging effects on students' learning in the simulation

Results were ambiguous concerning how emotions affected students' learning. Studies that focused on the learning process show how emotion can impact students' cognitive load and that stress and a high cognitive load can influence students' ability to learn (Fraser et al., 2012, 2014; Fraser & McLaughlin, 2018; Schlairet et al., 2015; Tremblay et al., 2017). Students who experience high cognitive load may miss important clinical observations (Schlairet et al., 2015). Stress limits their ability to focus and make it difficult to think and do at the same time; for example, students report forgetting new knowledge and memory loss as a result of stress during simulations.

The literature show that stress and anxiety can negatively influence learning outcomes (Cato, 2013; Fraser et al., 2012; Fraser & McLaughlin, 2018; Tremblay et al., 2017; Walton et al., 2011); for example, by impairing performance and the development of competency. However, stress and anxiety can also benefit learning outcomes. For example, Demaria et al. (2010, 2016) found that students exposed to emotionally challenging scenarios (e.g. by letting the manikin die) achieved greater practical competence than those who were not exposed to this scenario. Several studies found that moderate anxiety is experienced by students as a driver of learning (Al‐Ghareeb et al., 2019; Behrens et al., 2019; Groot et al., 2020; Ko & Choi, 2020). Five studies identified compound learning outcomes such as confidence and feeling more secure, despite the students being emotionally aroused. Students have reported that they gain experience by practising clinical reasoning and critical thinking, and by learning to identify errors, and that the simulation experience seems to motivate them to improve and to direct their own learning (Groot et al., 2020; Holt, 2017; Ko & Choi, 2020; Najjar et al., 2015; Tremblay et al., 2017; Walton et al., 2011). For example, during a simulation, students learn to analyse the patient's situation and to prioritize and implement interventions (Walton et al., 2011). Holt (2017) described complex affective learning outcomes, such as empathy and confronting ethical issues.

Ambiguous results were found in the mixed‐methods study by Beischel (2013). The quantitative aspect of their study found that anxiety does not influence the learning process, whereas the qualitative aspect found that anxiety prevents learning. Experience of anxiety causes slower thinking and has a negative effect on students' ability to think clearly. Walton et al. (2011) found that stress and anxiety can hinder the learning process even though the students reported complex learning outcomes.

## 4 DISCUSSION

Interestingly, our findings show that there is a lack of detailed knowledge about students' emotions during SBE. Our analysis showed that studies have focused mainly on students' reactions to the simulated learning experience, for example, anxiety about their performance in SBE. Emotional activation triggered by cognitive incongruity, SBE content and social interactions is rarely described in the literature. One of the most important findings of our analyses was that stress and anxiety are the predominant emotions reported in the literature and that these affect academic outcomes mainly in an obstructive way. However, experienced emotions are important for learning regardless of whether the experiences are pleasant or unpleasant.

### 4.1 The emotional rollercoaster during SBE

Learning situations foster intertwined mixed emotions (Shuman et al., 2013) and this also occurs in the simulation context. Our analysis showed that during simulation students express emotions ranging from fear to excitement, enjoyment and pride. Students have reported feeling confident, calm, surprised, shamed, frustrated, proud and happy at the end of a simulation (Behrens et al., 2019; Tremblay et al., 2017; Walton et al., 2011). These findings support the idea that emotions are dynamic and that students can experience both positive and negative emotions in the same educational context (D'Mello & Graesser, 2012; Shuman et al., 2013).

In the debriefing phase where learning mainly occurs, negative emotions can shift to positive if students are given the opportunity to reflect on the scenario situation – what worked, what did not work – and to obtain supportive feedback (Dufrene & Young, 2014). Such an emotional change can play an important role in students developing confidence about the skills needed to become a professional health‐care worker.

Our analysis also show that students can experience emotional overload during SBE. In our analysis overload was defined as a high cognitive load. A high cognitive load has been found to affect working memory capacity by limiting the processing of novel information and working memory (Van Merrienboer & Sweller, 2005). Studies have identified that high cognitive load during simulation can reduce performance and memory months after the simulation (Fraser et al., 2012; Fraser & McLaughlin, 2018; Schlairet et al., 2015) which suggest that a higher cognitive load can also impact learning outcomes.

### 4.2 Negative emotions are more than a barrier

Studies of emotions in education are dominated by studies of anxiety and stress (Pekrun & Linnenbrink‐Garcia, 2014). This is also the case in the literature examined in this review because several of the included studies described a one‐sided focus on simulation as a nerve‐wracking experience that is loaded with stress, fear, discomfort and anxiety. Such emotions are categorized as negative and unpleasant according to the CM.

Most of the studies in this review reported anxiety and stress as hampering learning (Cato, 2013; Fraser et al., 2012; Fraser & McLaughlin, 2018; Tremblay et al., 2017; Walton et al., 2011). In academic situations, anxiety is perceived as the most problematic emotion for academic success. Anxiety impacts the learner's ability to focus, which negatively impacts working memory. Research on anxiety in test situations supports the idea that negatively loaded experiences adversely influence cognitive processes by requiring use of the available cognitive resources to cope with anxiety, leaving few resources for memorizing, interpreting and solving complex tasks (Pekrun & Linnenbrink‐Garcia, 2014; Valiente et al., 2012). This is supported by the findings of our review, in which stress and anxiety were found to increase memory loss, decreased the ability to focus on the task, and hinder thinking (Cato, 2013; Walton et al., 2011). However, our results also found that moderate stress and anxiety have a positive effect and can facilitate students' learning because the anxiety associated with SBE improves students' preparedness (Behrens et al., 2019; Ko & Choi, 2020). Demaria et al. (2010) and Al‐Ghareeb et al. (2019) also found that moderate stress and low‐level anxiety were associated with greater practical competency and optimal performance. Incorporating some tension before a simulation, for example by requiring students to prepare in advanced, is supported by the simulation framework (Jeffries, 2020).

Walton et al. (2011), Behrens et al. (2019) and Holt (2017) found that students can feel confused and frustrated during the simulation. Students face confusion and frustration when experiencing incongruity between cognitive capacity and performance and when their existing knowledge is inconsistent with new information (Vogl et al., 2019). However, such unpleasant emotions can benefit learning. Most learners want to overcome the feeling of confusion, and to do so, they engage and seek knowledge to resolve the feeling of incongruity. Therefore, confusion can increase students' deeper level of understanding (Lehman et al., 2012). In particular the opportunity to resolve their confusion during a simulation activity broadens the potential for learning (Fredrickson, 2001). Through the debriefing phase, learners are given the opportunity to explore and discuss problems experienced in the simulation scenario.

Our analysis also showed that, despite the students' anxiety, stress and discomfort, complex learning outcomes do occur (Al‐Ghareeb et al., 2019; Demaria et al., 2010; Groot et al., 2020; Holt, 2017; Najjar et al., 2015; Walton et al., 2011). Students gain confidence and understanding, learn to analyse the patient's situation and increase their practical competency. According to the CM, both negative and positive emotions can activate students, and this activation can benefit the learning process by increasing attention, alertness, concentration and engagement in the learning task (Ainley et al., 2002). The results of our analysis show that students are highly emotionally activated during a simulation. Arousal can play a crucial role in students' learning and learning outcomes beyond the potential for pleasant or unpleasant emotions to interrupt the learning process.

### 4.3 Enjoyment and relaxation foster learning in a simulation

Students' positive emotions include excitement, pride and enjoyment (Behrens et al., 2019; Groot et al., 2020; Holt, 2017; Schlairet et al., 2015). These are defined as activated emotions in the CM (Posner et al., 2005). Positive activated emotions can foster interest, and the motivation to study, and can contribute to the desire for students to make the effort to explore new knowledge. Interest helps students to maintain attention in the academic setting. Sharpened attention, increased effort and interest are beneficial in the learning process (Fredrickson, 2001). By being in a positive state, students can feel free to think critically and solve problems in flexible and creative ways (Pekrun et al., 2002). Solving problems and seeking understanding are important in a simulation. These actions are related to metacognition and deep learning approaches, which are associated with higher achievement scores (Pekrun & Linnenbrink‐Garcia, 2014; Trigwell et al., 2012).

Students also described positive deactivated emotions, such as a “sigh of relief” and calmness after the scenario has ended (Fraser et al., 2012; Groot et al., 2020; Najjar et al., 2015; Schlairet et al., 2015), and these emotions are most prominent during debriefing (Fraser & McLaughlin, 2018). Students can benefit from being in a low‐arousal state before entering the debriefing phase. Being relieved and calm can make the space needed for cognition and reflection, which are important for understanding and learning during a simulation (Neill & Wotton, 2011).

### 4.4 Strengths and limitations

To ensure validity of our analysis, the research process involved four researchers, including a senior librarian for quality assurance of the database search. Our analysis was strengthened by the broad search conducted and the updated search to include the latest research.

Despite the common aims used in many studies, research on emotions and learning encompasses a complex field, and emotions have been investigated in various dimensions. Learning has been defined as an outcome or process, which meant that comparison between studies was problematic and complicated our conclusions. Many of the studies included were conducted with a small number of participants, thus, it may be premature to draw conclusions about the topic.

### 4.5 Relevance to educational practice

The usual recommendation for simulations is to reduce stress to increase the learning experiences (Al‐Ghareeb et al., 2017; Cantrell et al., 2017). Our findings indicate a more complex and nuanced picture; that is, there is a wider spectrum of emotions occurring in simulation than simply students' stress and anxiety. The black‐and‐white perspective that students' stress and anxiety can have a negative effect on learning may be misleading. Our analysis shows that simulation can trigger multiple and changing emotions. Educators should consider the full range of students' emotions when planning and performing SBE. The need to establish a psychologically safe learning environment is relevant to SBE theory and is important for reducing student's anxiety (Kolbe et al., 2020). However, educators should also consider using SBE to trigger comprehensive emotions that can benefit students' learning such as curiosity, interest and confusion. The emotional rollercoaster that students experience during simulations highlights the importance of time for reflection and clarification during debriefing. Not including adequate debriefing can mean that students leave the simulation with unresolved frustration and confusion. Therefore, debriefing should be considered as an essential part of SBE.

## 5 CONCLUSION

The main findings of our analysis were the strong focus on stress and anxiety in the literature and the broader spectrum of students' emotions uncovered. This review did not identify unilaterally empirical proof for promoting positively or negatively loaded emotions limiting students' learning during SBE. The role of arousal in learning needs further clarification. The benefits of experiencing a range of emotions during a simulation should not be overlooked because the change in students' emotions from fear and stress to enjoyment and less arousal can help to facilitate their sense of amazement and investigation. Students should be allowed the opportunity to express frustration and to resolve their confusion. This opportunity is best given during debriefing, which supports the idea that debriefing is essential for learning during SBE.

Our results disclosed that little attention has focused on the effects of positive emotions on learning. This coincides with research about academic emotions overall (Pekrun & Linnenbrink‐Garcia, 2014). The reasons for the lack of research may be that students often are unaware of their enjoyment of learning and the difficulties observing and measuring positive emotions.

Because emotions are a highly subjective experience, future research should include more qualitative research to understand more fully students' learning during simulation. Research on the impact of emotions on learning during simulation should focus on how emotions change throughout the simulation, how activated emotions affect learning and how to scaffold and implement simulation scenarios to trigger academic emotions such as curiosity, frustration and surprise.

## CONFLICT OF INTEREST

The authors declare that they have no conflict of interest.

## ETHICAL APPROVAL

All authors provided substantial contributions to study design, analysis and synthesis of data. All authors have agreed on the final version to be published.

## Data Availability

The data that support the findings of this study are available on request from the corresponding author.

## References

[nop21100-bib-0001] Ainley, M. , Hidi, S. , & Berndorff, D. (2002). Interest, learning, and the psychological processes that mediate their relationship. Journal of Educational Psychology, 94(3), 545. 10.1037/0022-0663.94.3.545

[nop21100-bib-0002] Al‐Ghareeb, A. Z. , Cooper, S. J. , & McKenna, L. G. (2017). Anxiety and clinical performance in simulated setting in undergraduate health professionals education: An integrative review. Clinical Simulation in Nursing, 13(10), 478–491. 10.1016/j.ecns.2017.05.015

[nop21100-bib-0003] Al‐Ghareeb, A. Z. , Cooper, S. J. , & McKenna, L. G. (2019). The influence of anxiety on student nurse performance in a simulated clinical setting: A mixed methods design. International Journal of Nursing Studies, 98, 57–66. 10.1016/j.ijnurstu.2019.06.006 31284161

[nop21100-bib-0004] Artino, A. R. , Holmboe, E. S. , & Durning, S. J. (2012). Can achievement emotions be used to better understand motivation, learning, and performance in medical education? Medical Teacher, 34(3), 240–244. 10.3109/0142159X.2012.643265 22364457

[nop21100-bib-0005] Barrett, L. F. (2004). Feelings or words? Understanding the content in self‐report ratings of experienced emotion. Journal of Personality and Social Psychology, 87(2), 266. 10.1037/0022-3514.87.2.266 15301632 PMC1351136

[nop21100-bib-0006] Barrett, L. F. , & Russell, J. (2014). The psychological construction of emotion. Guilford Publications.

[nop21100-bib-0007] Behrens, C. C. , Dolmans, D. H. , Gormley, G. J. , & Driessen, E. W. (2019). Exploring undergraduate students achievement emotions during ward round simulation: A mixed‐method study. BMC Medical Education, 19, 316. 10.1186/s12909-019-1753-1 31438939 PMC6704623

[nop21100-bib-0008] Beischel, K. P. (2013). Variables affecting learning in a simulation experience: A mixed methods study. Western Journal of Nursing Research, 35(2), 226–247. 10.1177/0193945911408444 21593285

[nop21100-bib-0009] Bethea, D. P. , Castillo, D. C. , & Harvison, N. (2014). Use of simulation in occupational therapy education: Way of the future? American Journal of Occupational Therapy, 68(Supplement_2), S32–S39. 10.5014/ajot.2014.012716 25397936

[nop21100-bib-0010] Biggs, J. B. , & Collis, K. F. (2014). Evaluating the quality of learning: The SOLO taxonomy (Structure of the Observed Learning Outcome). Academic Press.

[nop21100-bib-0011] Blackstock, F. C. , & Jull, G. A. (2007). High‐fidelity patient simulation in physiotherapy education. Australian Journal of Physiotherapy, 53(1), 3–5. 10.1016/S0004-9514(07)70056-9 17326733

[nop21100-bib-0012] Campbell, S. H. , & Daley, K. M. (2018). Simulation scenarios for nurse educators: Making it real (3rd ed.). Springer.

[nop21100-bib-0013] Cant, R. P. , & Cooper, S. J. (2010). Simulation‐based learning in nurse education: Systematic review. Oxford, UK. 10.1111/j.1365-2648.2009.05240.x 20423432

[nop21100-bib-0014] Cant, R. P. , & Cooper, S. J. (2017). Use of simulation‐based learning in undergraduate nurse education: An umbrella systematic review. Nurse Education Today, 49, 63–71. 10.1016/j.nedt.2016.11.015 27902949

[nop21100-bib-0015] Cantrell, M. L. , Meyer, S. L. , Mosack, V. , & Cantrell, M. L. (2017). Effects of simulation on nursing student stress: An integrative review. The Journal of Nursing Education, 56(3), 139–144. 10.3928/01484834-20170222-04 28263351

[nop21100-bib-0016] Cato, M. L. (2013). Nursing students anxiety in simulation settings: A mixed Metohdes study. Doctor of Education, Portland State University, Portland State University.

[nop21100-bib-0017] Demaria, S. Jr , Bryson, E. O. , Mooney, T. , Silverstein, J. H. , Reich, D. L. , Bodian, C. , & Levine, A. I. (2010). Adding emotional stressors to training in simulated cardiopulmonary arrest enhances participant performance. Medical Education, 44(10), 1006–1015. 10.1111/j.1365-2923.2010.03775.x 20880370

[nop21100-bib-0018] Demaria, S. , Silverman, E. R. , Lapidus, K. A. B. , Williams, C. H. , Spivack, J. , Levine, A. , & Goldberg, A. (2016). The impact of simulated patient death on medical students' stress response and learning of ACLS. Medical Teacher, 38(7), 730–737. 10.3109/0142159X.2016.1150986 27052665

[nop21100-bib-0019] D'Mello, S. , & Graesser, A. (2012). Dynamics of affective states during complex learning. Learning and Instruction, 22(2), 145–157. 10.1016/j.learninstruc.2011.10.001

[nop21100-bib-0020] Dufrene, C. , & Young, A. (2014). Successful debriefing—Best methods to achieve positive learning outcomes: A literature review. Nurse Education Today, 34(3), 372–376. 10.1016/j.nedt.2013.06.026 23890542

[nop21100-bib-0021] Fraser, K. , Huffman, K. , Ma, I. , Sobczak, M. , McIlwrick, J. , Wright, B. , & McLaughlin, K. (2014). The emotional and cognitive impact of unexpected simulated patient death: A randomized controlled trial. Chest, 145(5), 958–963. 10.1378/chest.13-0987 24158305

[nop21100-bib-0022] Fraser, K. , Ma, I. , Teteris, E. , Baxter, H. , Wright, B. , & McLaughlin, K. (2012). Emotion, cognitive load and learning outcomes during simulation training. Medical Education, 46(11), 1055–1062. 10.1111/j.1365-2923.2012.04355.x 23078682

[nop21100-bib-0023] Fraser, K. , & McLaughlin, K. (2018). Temporal pattern of emotions and cognitive load during simulation training and debriefing. Medical Teacher, 41(2), 184–189. 10.1080/0142159X.2018.1459531 29687734

[nop21100-bib-0024] Fredrickson, B. L. (2001). The role of positive emotions in positive psychology: The broaden‐and‐build theory of positive emotions. American Psychologist, 56(3), 218–226. 10.1037/0003-066X.56.3.218 PMC3122271 11315248

[nop21100-bib-0025] Gluck, M. A. , Mercado, E. , & Myers, C. E. (2016). Learning and memory. From brain to behaviour. Worth Publishers.

[nop21100-bib-0026] Groot, F. , Jonker, G. , Rinia, M. , Cate, O. , & Hoff, R. G. (2020). Simulation at the frontier of the zone of proximal development: A test in acute care for inexperienced learners. Academic Medicine, 95(7), 1098–1105. 10.1097/ACM.0000000000003265 32134783

[nop21100-bib-0027] Harder, B. N. (2010). Use of simulation in teaching and learning in health sciences: A systematic review. Nursing Education, 49(1), 23–28. 10.3928/01484834-20090828-08 19731886

[nop21100-bib-0028] Hattingh, H. , Robinson, D. , & Kelly, A. (2018). Evaluation of a simulation‐based hospital pharmacy training package for pharmacy students. International Journal of Educational Technology in Higher Education, 15(1), 1–15. 10.1186/s41239-018-0120-3

[nop21100-bib-0029] Hegland, P. A. , Aarlie, H. , Strømme, H. , & Jamtvedt, G. (2017). Simulation‐based training for nurses: Systematic review and meta‐analysis. Nurse Education Today, 54, 6–20. 10.1016/j.nedt.2017.04.004 28456053

[nop21100-bib-0030] Holt, K. M. (2017). Affective domain learning in high fidelity simulation: Students' perspectives. Doctor of Philosophy, College of Natural and Health Sciences School of Nursing. Nursing Education, University of Northern Colorado.

[nop21100-bib-0031] Husebo, S. E. , Dieckmann, P. , Rystedt, H. , Soreide, E. , & Friberg, F. (2013). The relationship between facilitators' questions and the level of reflection in postsimulation debriefing. Simulation in Healthcare: The Journal of the Society for Simulation in Healthcare, 8(3), 135–142. 10.1097/SIH.0b013e31827cbb5c 23343839

[nop21100-bib-0032] Illeris, K. (2015). The development of a comprehensive and coherent theory of learning. European Journal of Education, 50(1), 29–40. 10.1111/ejed.12103

[nop21100-bib-0033] Issenberg, S. , William, C. , Petrusa, E. R. , Gordon, D. L. , & Scalese, R. J. (2005). Features and uses of high‐fidelity medical simulations that lead to effective learning: A BEME systematic review. Medical Teacher, 27(1), 10–28. 10.1080/01421590500046924 16147767

[nop21100-bib-0034] Jeffries, P. (2020). Simulation in nursing education: From conceptualization to evaluation. Lippincott Williams & Wilkins.

[nop21100-bib-0035] Keskitalo, T. , & Ruokamo, H. (2017). Students emotion in simulation‐based medical education. Journal of Interactive Learning Research, 28(2), 149–159.

[nop21100-bib-0036] Ko, E. , & Choi, Y.‐J. (2020). Debriefing model for psychological safety in nursing simulations: A qualitative study. International Journal of Environmental Research and Public Health, 17, 2826. 10.3390/ijerph17082826 PMC7215814 32325983

[nop21100-bib-0037] Kolbe, M. , Eppich, W. , Rudolph, J. , Meguerdichian, M. , Catena, H. , & Cripps, A. (2020). Managing psychological safety in debrifings: A dynamic balancing act. BMJ Simulation and Technology Enhanced Learning, 6(3), 164–171. 10.1136/bmjstel-2019-000470 PMC8936758 35518370

[nop21100-bib-0038] LeBlanc, V. R. , McConnell, M. M. , & Monteiro, S. D. (2015). Predictable chaos: A review of the effects of emotions on attention, memory and decision making. Advances in Health Sciences Education, 20(1), 265–282. 10.1007/s10459-014-9516-6 24903583

[nop21100-bib-0039] Lehman, B. , D'Mello, S. , & Graesser, A. (2012). Confusion and complex learning during interactions with computer learning environments. The Internet and Higher Education, 15(3), 184–194. 10.1016/j.iheduc.2012.01.002

[nop21100-bib-0040] Levett‐Jones, T. , & Lapkin, S. (2014). A systematic review of the effectiveness of simulation debriefing in health professional education. Nurse Education Today, 34(6), e58–e63. 10.1016/j.nedt.2013.09.020 24169444

[nop21100-bib-0041] Levine, A. I. , DeMaria, S. Jr , & Schwartz (2013). The comprehensive textbook of healthcare simulation. Springer.

[nop21100-bib-0042] Motola, I. , Devine, L. A. , Chung, H. S. , Sullivan, J. E. , & Issenberg, S. B. (2013). Simulation in healthcare education: A best evidence practical guide. AMEE Guide No. 82. Medical Teacher, 35(10), 1511–1530. 10.3109/0142159X.2013.818632 23941678

[nop21100-bib-0043] Najjar, R. H. , Lyman, B. , & Miehl, N. (2015). Nursing students' experiences with high‐fidelity simulation. International Journal of Nursing Education Scholarship, 12(1), 27–35. 10.1515/ijnes-2015-0010 25803087

[nop21100-bib-0044] Neill, M. A. , & Wotton, K. (2011). High‐fidelity simulation debriefing in nursing education: A literature review. Clinical Simulation in Nursing, 7(5), e161–e168. 10.1016/j.ecns.2011.02.001

[nop21100-bib-0045] Noyes, J. , Booth, A. , Moore, G. , Flemming, K. , Tunçalp, Ö. , & Shakibazadeh, E. (2019). Synthesising quantitative and qualitative evidence to inform guidelines on complex interventions: Clarifying the purposes, designs and outlining some methods. BMJ Global Health, 4(Suppl 1), e000893. 10.1136/bmjgh-2018-000893 PMC6350750 30775016

[nop21100-bib-0046] Olaussen, C. , Heggdal, K. , & Raaen, T. (2020). Elements in scenario‐based education associated with nursing students self‐confidence and satisfaction: A cross‐sectional study. Nursing Open, 7, 170–179. 10.1002/nop2.375 31871700 PMC6917966

[nop21100-bib-0061] Pekrun, R. , Goetz, W. , & Titz, P. P. (2002). Academic Emotions in Students' Self‐Regulated Learning and Achievement: A Program of Qualitative and Quantitative Research. Educational Psychologist, 37(2), 91–105. 10.1207/S15326985EP3702_4

[nop21100-bib-0047] Pekrun, R. (2019). Inquiry on emotions in higher education: Progress and open problems. Studies in Higher Education, 44(10), 1806–1811. 10.1080/03075079.2019.1665335

[nop21100-bib-0048] Pekrun, R. , & Linnenbrink‐Garcia, L. (2014). International handbook of emotions in education. Routledge.

[nop21100-bib-0049] Perry, S. , Bridges, S. M. , & Burrow, M. F. (2015). A review of the use of simulation in dental education. Simulation in Healthcare, 10(1), 31–37. 10.1097/SIH.0000000000000059 25574865

[nop21100-bib-0050] Posner, J. , Russell, J. A. , & Peterson, B. S. (2005). The circumplex model of affect: An integrative approach to affective neuroscience, cognitive development, and psychopathology. Development and Psychopathology, 17(3), 715–734. 10.1017/S0954579405050340 16262989 PMC2367156

[nop21100-bib-0051] Roth, W.‐M. , & Walshaw, M. (2019). Affect and emotions in mathematics education: Toward a holistic psychology of mathematics education. Educational Studies in Mathematics, 102(1), 111–125. 10.1007/s10649-019-09899-2

[nop21100-bib-0052] Schlairet, M. C. , Schlairet, T. J. , Sauls, D. H. , & Bellflowers, L. (2015). Cognitive load, emotion, and performance in high‐fidelity simulation among beginning nursing students: A pilot study. The Journal of Nursing Education, 54(3 Suppl), 5–11. 10.3928/01484834-20150218-10 25692940

[nop21100-bib-0053] Shuman, V. , Sander, D. , & Scherer, K. R. (2013). Levels of valence. Frontiers in Psychology, 4, 261. 10.3389/fpsyg.2013.00261 23717292 PMC3651968

[nop21100-bib-0060] Trigwell, K. , Ellis, R. A. , & Han, F. (2012). Relations between students' approaches to learning, experienced emotions and outcomes of learning. Studies in Higher Education, 37(7), 811–824.

[nop21100-bib-0054] Tremblay, M. L. , Lafleur, A. , Leppink, J. , & Dolmans, D. (2017). The simulated clinical environment: Cognitive and emotional impact among undergraduates. Medical Teacher, 39(2), 181–187. 10.1080/0142159X.2016.1246710 27832706

[nop21100-bib-0055] Valiente, C. , Swanson, J. , & Eisenberg, N. (2012). Linking students' emotions and academic achievement: When and why emotions matter. Child Development Perspectives, 6(2), 129–135. 10.1111/j.1750-8606.2011.00192.x 23115577 PMC3482624

[nop21100-bib-0056] Van Merrienboer, J. , & Sweller, J. (2005). Cognitive load theory and complex learning: Recent developments and future directions. Educational Psychology Review, 17(2), 147–177. 10.1007/s10648-005-3951-0

[nop21100-bib-0057] Vogl, E. , Pekrun, R. , Murayama, K. , & Loderer, K. (2019). Surprised–curious–confused: Epistemic emotions and knowledge exploration. Emotion, 20(4), 625–641. 10.1037/emo0000578 30883147

[nop21100-bib-0058] Walton, J. , Chute, E. , & Ball, L. (2011). Negotiating the role of the professional nurse: The pedagogy of simulation: A grounded theory study. Journal of Professional Nursing, 27(5), 299–310. 10.1016/j.profnurs.2011.04.005 21925463

[nop21100-bib-0059] Whittemore, R. , & Knafl, K. (2005). The integrative review: Updated methodology. Journal of Advanced Nursing, 52(5), 546–553. 10.1111/j.1365-2648.2005.03621.x 16268861

